# Sacrificial Cladding with Brittle Materials for Blast Protection

**DOI:** 10.3390/ma14143980

**Published:** 2021-07-16

**Authors:** Ludovic Blanc, Thérèse Schunck, Dominique Eckenfels

**Affiliations:** French-German Research Institute of Saint-Louis, 5 Rue du Général Cassagnou, CEDEX, 68300 Saint-Louis, France; therese.schunck@isl.eu (T.S.); dominique.eckenfels@isl.eu (D.E.)

**Keywords:** experimental work, blast protection, sacrificial cladding, brittle deformation

## Abstract

In the following work, sacrificial claddings filled with different brittle materials were investigated, from concrete foam to granular media. They were subjected to blast loading using an explosive driven shock tube, while a sensor measures the load transmission and a high speed camera records the compression of the core. From a macroscopic point of view, concrete foam and granular media can act efficiently as a crushable core but differs greatly in terms of energy dissipation mechanisms. To compare them, granular media was at first treated as a cellular material, and different key parameters (plateau stress, densification strain) were computed using the energy absorption efficiency methodology. The presented tests results, coupled with observation in literature, allow a better understanding on the crushing process of a granular media. In particular, granular media tend to work as a core even for low intensity load, contrary to more classical crushable core.

## 1. Introduction

**Figure 1:**
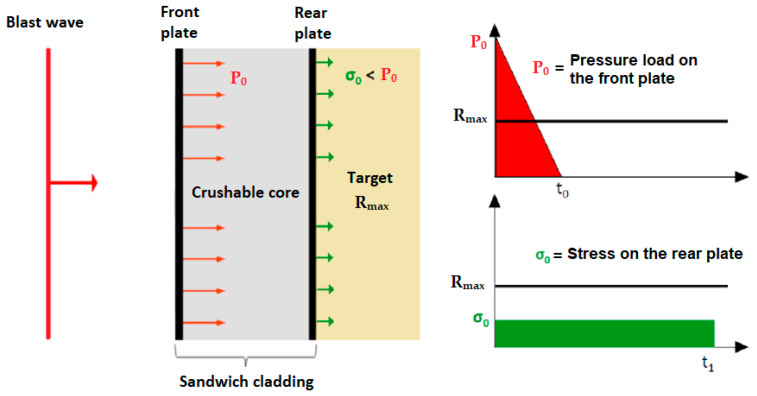
Sacrificial cladding submitted to an ideal planar shock wave.

The general principle of blast protection consists in placing an energy absorbing material between the explosive charge and the target. Depending on its position, this material aims to mitigate the detonation of the explosive [1,2], to disturb the blast wave propagation [3], or to passively protect the target [4]. Among these solutions, sacrificial claddings are passive architectures which allow to dissipate the blast wave energy and to improve the target’s resistance against such solicitation [4,5,6,7]. It is made of three components: a crushable core, sandwiched between a front plate and a rear plate. When submitted to a blast wave, the front plate is put into displacement and crushes the core. Elastic, plastic, and brittle deformation of the core leads to energy dissipation and the transmission of a lower, quasi-constant loading over a longer time span (Figure 1).

**Figure 2:**
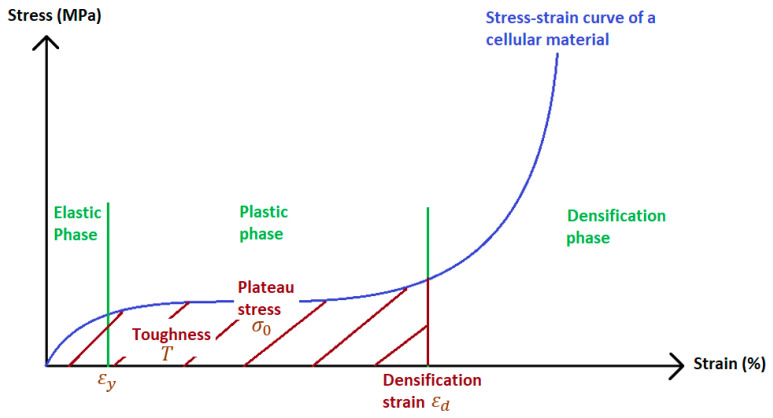
Ideal stress–strain curve of a cellular material.

The efficiency of a sacrificial cladding is greatly dependent on its three components. The front plate will lead the fluid-structure interaction between the blast wave and the architecture [8] while the core will lead the energy absorption mechanisms [9]. For that last reason, numerous studies with different materials were performed, especially with cellular materials. A cellular material is defined as a material made up of an interconnected network of solid struts or plates which form the edges and faces of cells [10]. The energy absorption capability of cellular materials has already been widely described in literature [9,11]. The generic stress–strain curve presented on Figure 2 shows the ideal behavior of these materials under compression.

This behavior is defined by three phases: the elastic phase, the plastic phase where the stress remains constant as the strain increases, and the densification phase where, because the edges of the cells come in contact, the compression is stopped and the stress rises significantly. To describe this curve, three parameters are important:

- Material toughness T: integration of the stress/strain evolution corresponding to the energy dissipated by the cellular material under compression;
- Plateau Stress σ_0_: quasi constant transmitted load between the end of the elastic phase of the material ε_y_ and the beginning of its densification phase ε_d_;
- Densification strain ε_d_: strain at which the struts and plates of the cellular material come into contact, leading to the densification of the sample. It provides information on the thickness necessary to avoid the rise of the stress during the densification process. P_0_.

These three parameters were determined by many authors for many materials using quasi-static and dynamic compression test: polymer foam [12,13,14], metal foam [4,15,16], hybrid foam [17], honeycomb [6,18], and tubular structures [19,20]. All of these materials are traditionally used energy absorbers in sacrificial cladding due to their elastic and plastic properties. On the contrary, brittle cellular materials and granular media have almost never been used in a sacrificial cladding, despite having the potential for energy dissipation. To the best of the author’s knowledge, while numerous studies detail the behavior of concrete foam under compression [21], studies involving claddings filled with concrete foam are limited to [22,23] while claddings filled with granular media (pumice and perlite) has been scarcely investigated [24].

In this paper, sacrificial claddings will be investigated using an explosive driven shock tube (EDST). The macroscopic behavior of four different granular media (perlite, clay, pumice, and hydrogel) will be compared with the macroscopic behavior of two cellular materials (a concrete foam and an aluminum honeycomb). For comparison purpose, the classical definition of the densification used for cellular materials will be extend to granular media by assuming that the densification is reached once the grains cannot be moved or crushed anymore. The samples have been chosen to exacerbate the differences in phenomenology between the granular and cellular materials. Based on the results, the efficiency of granular materials in a sacrificial cladding will be established.

## 2. Materials and Methods

### 2.1. Methodology

**Figure 3:**
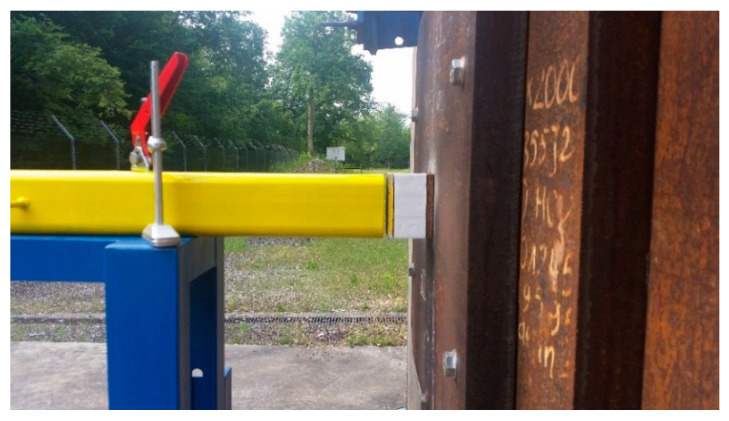
Pictures of the experimental set-up.

Planar shocks were generated with an explosive driven shock tube, following the methodology developed by Ousji [25]. This experimental set-up is ideal when it comes to studying the behavior of an architecture submitted to a blast loading. The shock tube has a square 100 × 100 mm^2^ external and 80 × 80 mm^2^ internal section *A*, for a 1750 mm total length *L*, and was placed against a rigid bunker, as shown on Figure 3.

**Figure 4:**
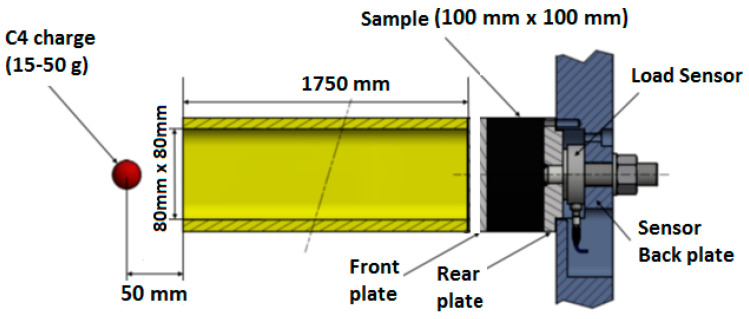
Schematic view of the explosive driven shock tube and load sensor.

As an initial condition, explosive charges (from 15 to 50 g C4) were detonated five centimeters in front of the EDST. As shown on Figure 4, the sacrificial cladding is placed between the tube and the bunker, with a small gap between the tube and the front plate to stop the shock transmission from the tube to the architecture’s front plate. There is no confinement of the crushable core, and no restriction on the front plate’s displacement apart from the reaction of the core. The steel front plate dimension is 100 × 100 × 8 mm^3^ and weight M_plate_ = 652 g A PCB 206C quartz force ring sensor is placed between the back plate and the bunker side wall as shown on Figure 4. It is not possible to increase the instrumentation as any sensor added to the system would be damaged by the blast (an optimal sensor positioning has not been researched [26]). The back plate fixed on the chassis of the bunker is considered rigid. The level of electronic noise is negligible in regard to the blast and transmitted load signals.

**Figure 5:**
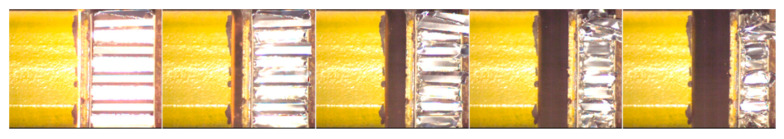
Pictures of the honeycomb during the crushing process.

Each test was recorded using a Phantom V311 high-speed camera set-up 0.5 m away from the end of the tube. The characteristics are the following: f85 lens, 1.4 aperture, exposure time lower than 8 μs which guarantees a sufficient luminosity during the recording, frame rate at 31 kfps, with a total recording time of 2 s providing an adequate time discretization to measure the displacement of the front plate (Figure 5).

In order to estimate the bare load at the end of the EDST, the signal was first measured without any sandwiched cladding. Tests were performed to estimate both the incident and reflected shock parameter at the end of the tube (respectively, incident and reflected overpressure Δ*P_i_* and Δ*P_r_*, incident and reflected impulse *I_i_* and *I_r_*, incident and reflected phase duration *t_i_* and *t_r_*). The incident parameters were used to compare the load with a free-field detonation of TNT.

For the incident shock, the results are:

- for 15 g of C4: Δ*P_i_* = 7.99 bars, *I_i_* = 3.09 bars.ms, *t_i_* = 1.28 ms. Based on the UFC 3-340-02 [27], these effects are equivalent to those of 17.3 kg of TNT placed 2.8 m away from the target;
- for 30 g of C4: Δ*P_i_* = 14.29 bars, *I_i_* = 5.15 bars.ms, *t_i_* = 1.52 ms. These effects are equivalent to those of 87 kg of TNT placed 3.3 m away from the target;
- for 50 g of C4: Δ*P_i_* = 22.53 bars, *I_i_* = 7.42 bars.ms, *t_i_* = 1.61 ms. These effects are equivalent to those of 567 kg of TNT placed 4.72 m away from the target.

**Figure 6:**
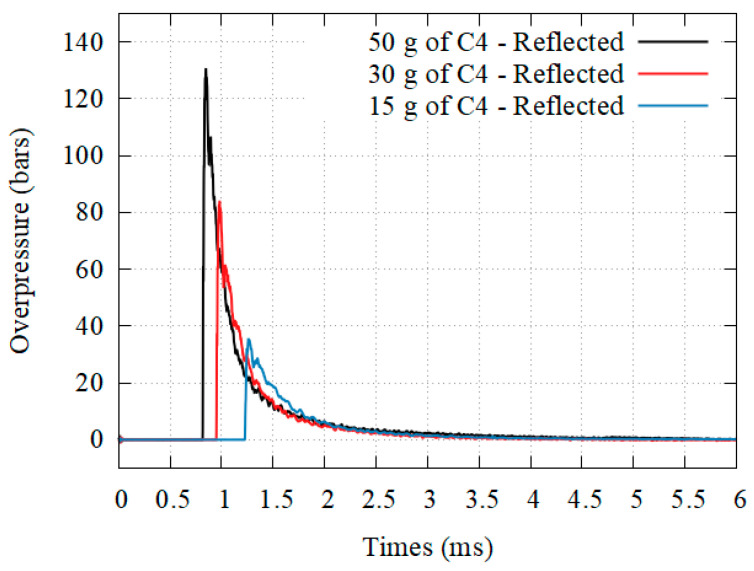
Reflected loading profiles: Overpressure.

For the reflected shock, the load sensor was replaced with a Kulite HKS-375 pressure transducer. The results are shown Figure 6:

- for 15 g of C4: Δ*P_r_* = 38.72 bars, *I_r_* = 16.98 bars.ms, *t_r_* = 3.22 ms;
- for 30 g of C4: Δ*P_r_* = 79.69 bars, *I_r_* = 26.54 bars.ms, *t_r_* = 3.36 ms;
- for 50 g of C4: Δ*P_r_* = 131.55 bars, *I_r_* = 39.76 bars.ms, *t_r_* = 3.42 ms.

To fully analyze the experimental data measured with the sandwich cladding, several steps are necessary:

- With the sensor, the load versus time curve *F*(*t*) can be plotted, and with the video recording, the time of maximum front plate displacement is known. The transmitted impulse *I_trans_* to the target can be calculated with Equation (1) and can be compared with the transmitted impulse to the front plate:

(1) $$I_{trans}=\int_{0}^{t_{end}}F(t)dt$$

In the same manner, the transmitted load to the target can be compared with the transmitted load to the front plate.

- The load versus time curve *F*(*t*) gained from the sensor, and the displacement versus time curve *l*(*t*) gained from the video recording, are cross-referenced in order to calculate the load-displacement curve *F*(*l*) of the material. Knowing the surface *A_sample_* and thickness *h_sample_* of the sample, it can be converted into the stress–strain curve *σ*(*ε*) similar to the one shown Figure 2.
- The energy *E_abs_* absorbed by the sample before reaching the densification is calculated using Equation (2).

(2) $$E_{abs}=\;\int_{0}^{l_{\max}}F(l)dl\;$$

**Figure 7:**
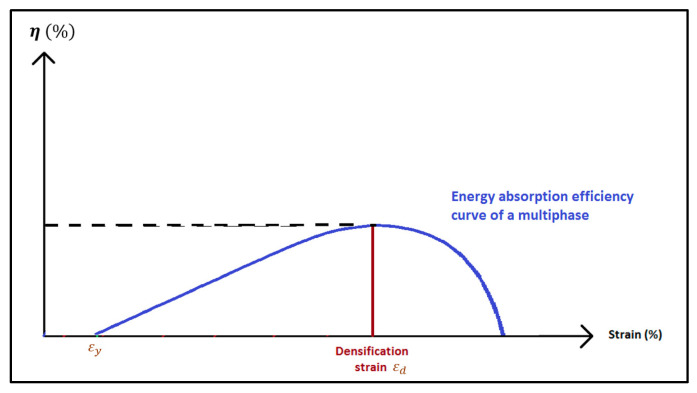
Energy efficiency absorption curve *η*(*ε*).

- The densification strain *ε_d_* is calculated based on the development chosen by Li [28]. It is defined as the point where the energy absorption efficiency *η*(*ε*), given in Equation (2), reaches a maximum on the efficiency–strain curve (Figure 7).

(3) $$\eta (\varepsilon )=\frac{1}{\sigma (\varepsilon )}\int_{\varepsilon_{y}}^{\varepsilon }\sigma (\varepsilon )d\varepsilon$$

The plateau stress value *σ_0_* is then calculated using the Equation (3). With *ε_y_* being the strain at which the plastic behavior starts:

(4) $$\sigma_{0}=\frac{\int_{\varepsilon_{y}}^{\varepsilon_{d}}\sigma (\varepsilon )d\varepsilon }{\varepsilon_{d}-\varepsilon_{y}}$$

This calculation is not possible if the densification phase is not reached. In that case, we will only have a lower limit for the densification strain and the plateau stress should be directly estimated on the curve.

- The toughness *T* of the material can then be calculated with Equation (4). By definition, this is the total energy per unit volume a sample can dissipate before densification.

(5) $$T\;=\;\int_{0}^{\varepsilon_{d}}\sigma (\varepsilon )d\varepsilon \;\approx \;\sigma_{0}\cdot \varepsilon_{d}$$

There are three pieces of information that must be kept in mind:

- Firstly, all of the material parameters calculated above are strain-rate dependent. It has been shown in the literature that for most materials, the higher the strain-rate, the higher the plateau stress and the lower the densification strain.
- Secondly, this experimental set-up only allows to study the macroscopic behavior of the core, hence this analytical approach. While it is not originally designed for granular materials, it is assumed that the deformation and densification of the granular media will be similar to those of cellular materials and the following methodology should be applied. To the best of the author‘s knowledge, there is no alternative at this time.
- Lastly, it is not possible to know prior to the test the quantity of energy transmitted to the core. In a sacrificial cladding, the blast wave energy is converted into kinetic energy which is then dissipated by the core. However, the energy transmitted to the system is dependent on the system itself. Since the front plate is not projected on the core, a stronger core will react strongly against the displacement of the front plate and limit its acceleration. In fine, this experimental set-up allows the characterization of a system composed of the front plate and the core, but does not allow the characterization of the core alone, as a drop tower would do. This peculiar approach has already been used for investigating blast protection because it allows to take into account the full phenomenology of the blast interaction with the structure and the blast loading profile [4,7,24], making it closer to practical application. However, this set-up could be used more traditionally by projecting the front plate on the sample, like a horizontal drop tower focusing on velocity instead of weight. Such approach is not discussed in this paper but can be used to estimate the maximal kinetic energy which could be transfer to the core. For a 652 g steel plate, its maximal kinetic energy under a 50 g solicitation would be close to 476 J.

### 2.2. Test Samples

**Figure 8:**
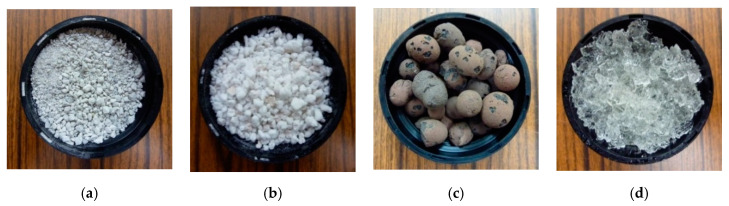
Pictures of core samples: (**a**) pumice; (**b**) perlite: (**c**) clay balls; (**d**) hydrogel.

Several cores were investigated in our laboratory, by putting them inside a sandwich cladding:

- Aluminum Hexagonal Honeycomb (ρ = 40 kg/m^3^): this core acts as a reference due to its inherent efficiency for energy dissipation through plastic deformation.
- Pumice (ρ = 910 kg/m^3^), Perlite (ρ = 120 kg/m^3^), and Clay Ball (ρ = 680 kg/m^3^): presented Figure 8, these granular media are able to act as a potential crushable core thanks to the brittle deformations and displacement of their grains.
- Hydrogel (ρ = 616 kg/m^3^): the polymer consists of 10% of Crosslinked Copolymer Acrylamide—Potassium Acrylate powder (APRODEV™ 06, APROTEK, Saint-Romain-le-Puy, France) and 90% water. In comparison to the previous materials, this granular media should present an elasto-plastic behavior.
- Concrete foam (from 150 to 700 kg/m^3^): this brittle cellular material is compared with the previous brittle granular materials.

**Figure 9:**
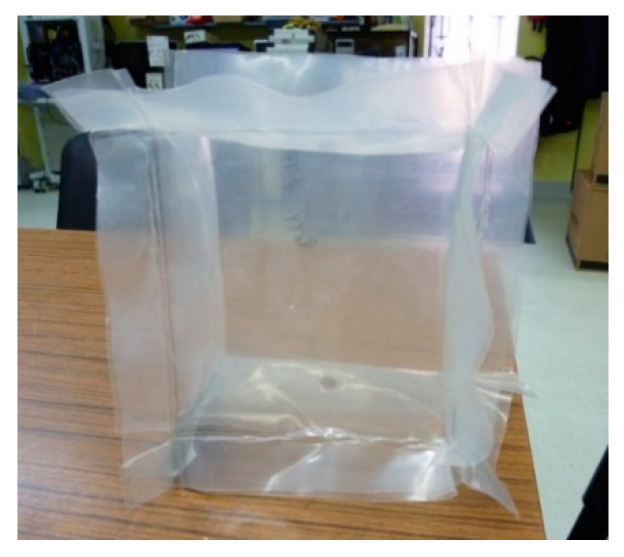
Unfilled squared plastic bags.

To hold the energy dissipative material in place until the beginning of the solicitation, the granular materials were encased in a 50 mm thick squared plastic bags (100 mm × 100 mm) (Figure 9).

## 3. Results

**Figure 10:**
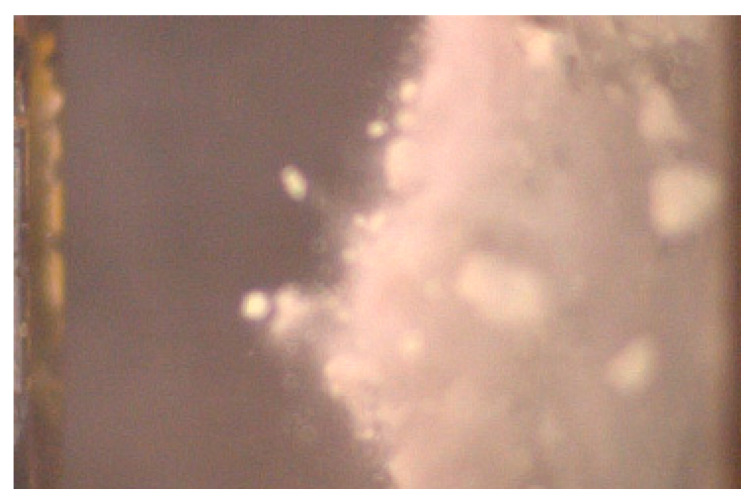
Concrete dust for the 150 kg·m^−3^ configuration: the front plate cannot be tracked anymore.

Three different type of results have been recorded. The sample is either fully crushed (densification), slightly crushed (no densification), or not crushed (no displacement of the front plate). The densification is necessary to compute all of the material parameters. When the densification is not reached, an underestimation of the parameters is given based on the last known position of the front plate. For four configurations, hydrogel and concrete foam, the dust and grains displacement was such that the front plate was not visible during the process. It can be assumed that the densification is directly linked to the quantity of materials expulsed from the system, hence to the accuracy of the video recording (Figure 10): there is simply more dust when the sample is pulverized.

Table 1 sums up the parameters gained from the test.

### 3.1. Honeycomb

**Figure 11:**
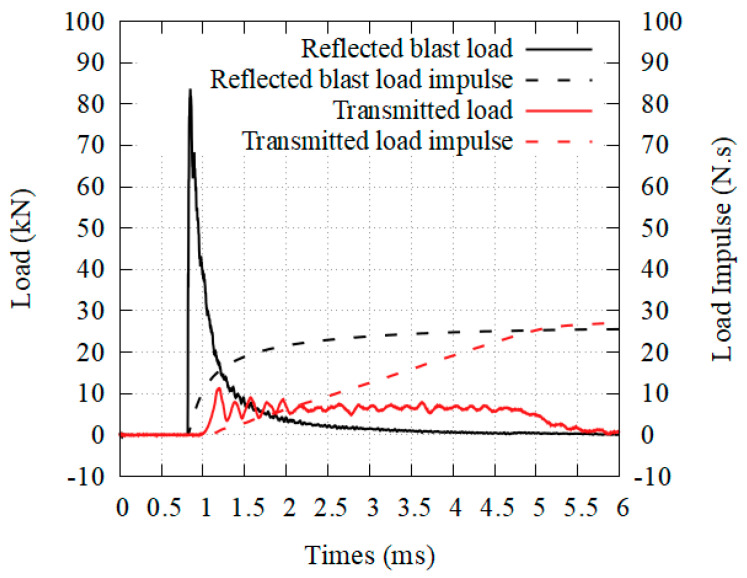
Reference transmitted load and load impulse with honeycomb.

Figure 11 presents a reference trial performed with honeycomb, where densification was avoided. As can be seen, there is a “conversion” of the initial blast loading into a quasi-constant lower load over a longer time span. It may seem that the impulse is conserved throughout the phenomenon but the momentum transmission, starting from the blast interaction with the front plate and finishing with the crushing of the core, is far more complex to look at. It generally involves FSI-reduction (which can be neglected in all of the presented tests due to the weight of the front plate), inertia and micro-inertia effects, pneumatic effect, and will not be discussed in this paper.

### 3.2. Concrete Foam

**Figure 12:**
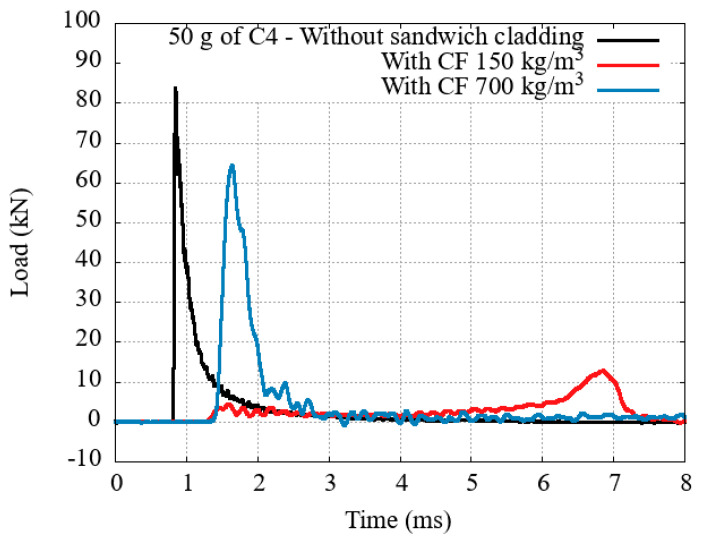
Transmitted load with concrete foams of two different densities.

Two different densities of 100 × 100 × 100 mm^3^ concrete foam were investigated: 150 and 700 kg·m^−3^. Their behavior is reported in Figure 12.

**Figure 13:**
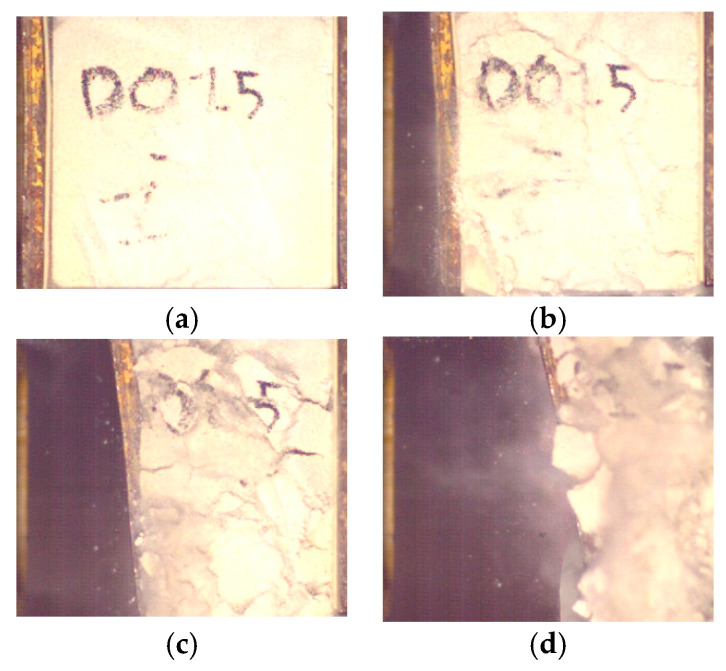
Low density sample fracturing process at 0 ms (**a**), 1.5 ms (**b**), 2.5 ms (**c**) and 5 ms (**d**).

**Figure 14:**
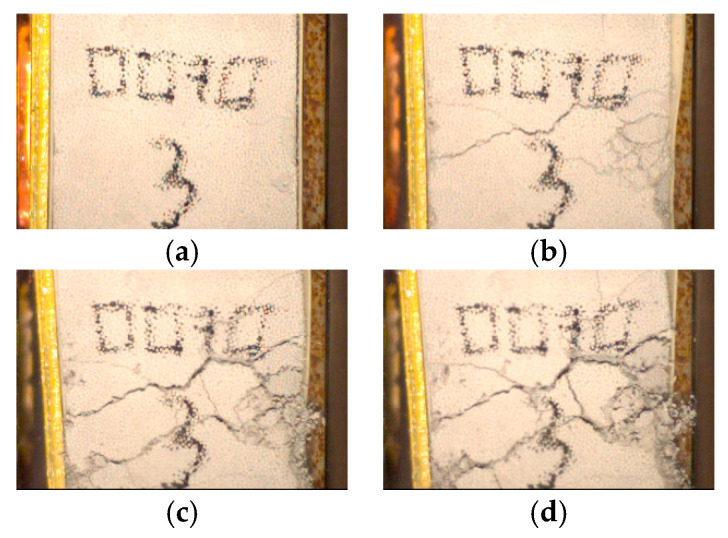
High density sample fracturing process at 0 ms (**a**), 1.5 ms (**b**), 2.5 ms (**c**) and 5 ms (**d**).

The difference in behavior is directly linked to the physical state of the sample after the test. The low density sample is pulverized (Figure 13), while a few fractures are visible on the high density sample (Figure 14).

- On the low density sample, the applied forced is enough to start the fractures near the front plate. The sample weakens, decreases its reaction against the front plate displacement. From this point onward, the concrete foam is crushed progressively, dissipating a total of 231 J. A slight densification near a 90% strain is estimated. Compared to honeycomb, this value is particularly high, and can be explained by the concrete dust and chunks expulsed from under the plate. This is not possible with an elasto-plastic material, where most of the material of the deformed sample will act against the displacement of the front plate.
- On the contrary, the collapse stress on the densest sample is not reached with the applied solicitation. A compressive wave is sent through the sample and is reflected at its end against the rigid wall, leading to an increase of the stress at the bottom of the sample. This stress is high enough to start fracturing the sample from its end, but is insufficient to completely crush the sample.

**Figure 15:**
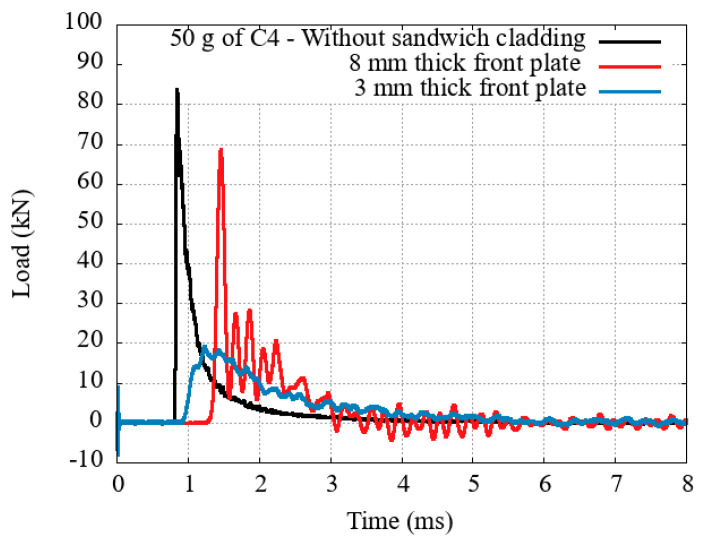
Transmitted load for a concrete foam of intermediate density (400 kg·m^−3^), two different solicitations.

An intermediate density of 400 kg·m^−3^ was investigated. The first test presented a behavior similar to the high density sample, with an almost complete transmission of the load. The solicitation was then increased by choosing a lighter front plate. This process allows to increase the kinetic energy of the front plate and to increase the load transmitted to the sample. This was enough to crush the sample but not enough to reach the densification (Figure 15). In the following discussion concrete foam will be shown to behave similarly to any cellular materials.

### 3.3. Brittle Granular Materials

**Figure 16:**
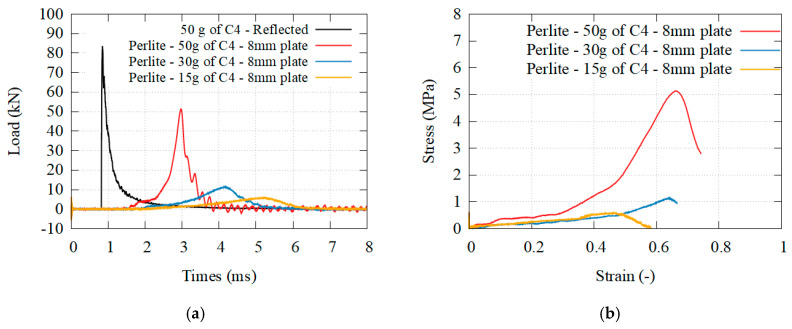
(**a**) Transmitted load for granular perlite at three different solicitations; (**b**) equivalent stress–strain curves.

**Figure 17:**
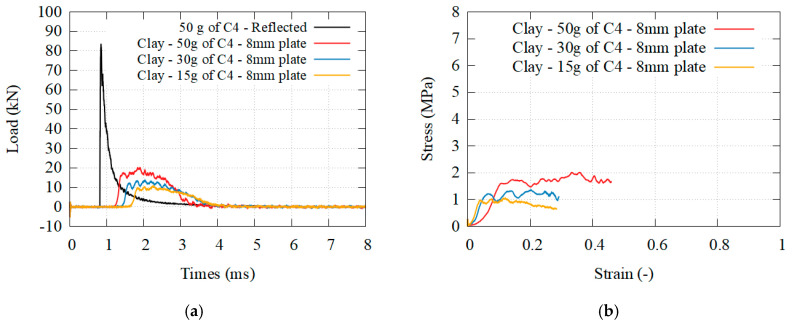
(**a**) Transmitted load for granular clay at three different solicitations; (**b**) equivalent stress–strain curves.

**Figure 18:**
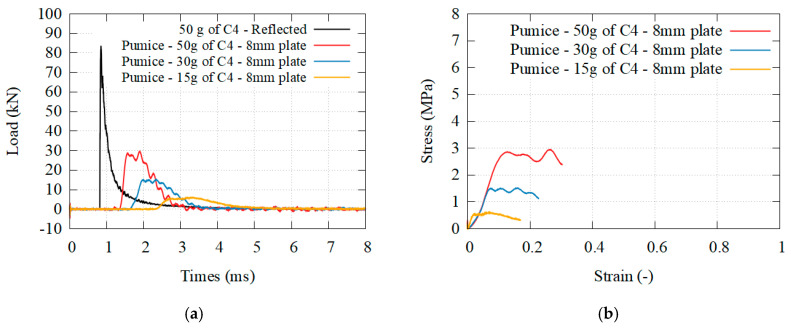
(**a**) Transmitted load for granular pumice at three different solicitations; (**b**) equivalent stress–strain curves.

**Figure 19:**
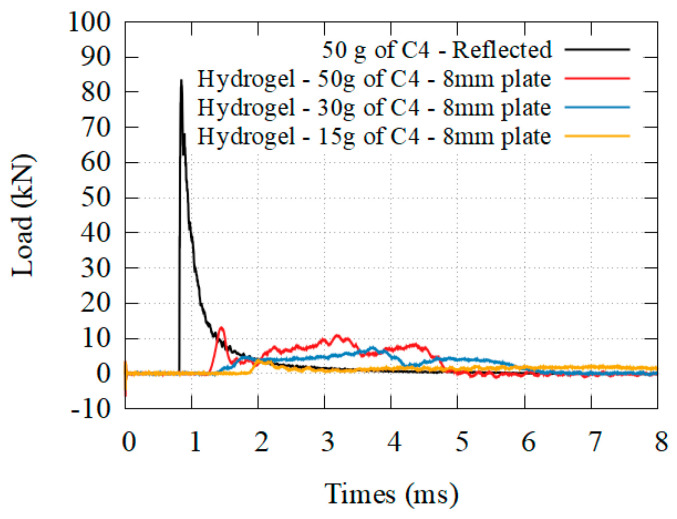
Transmitted load for granular hydrogel at three different solicitations.

Figure 16, Figure 17 and Figure 18 presents the transmitted load behind different granular core, for different solicitations, as well as the beginning of the stress–strain curve. Figure 19 presents the transmitted load behind hydrogel. The densification was reached only for the perlite. The higher the density, the higher the plateau stress of the granular media. The higher the solicitation, the higher the plateau stress. A complete analysis is given in the discussion.

## 4. Discussion

The dissipative properties of brittle materials in sandwich cladding can be observed in the previous section. The materials was chosen to investigate both the effect of the fragile constituting material (brittle behavior of a grain, of the concrete foam) and compare this effect with the degree of order of the material (stochastic foam, granular material).

The potential of concrete foam in sandwich cladding has been confirmed. A concrete foam behaves similarly to any kind of stochastic foam, or usual crushable core: it is a solid architecture, which collapses only if the brittle collapse stress is reached. This was already established by Tian [23], whose study focused on the deflection of large panel filled with concrete foam. In his paper, every sample was chosen so that the concrete foams were crushed, not deviating from the sacrificial design concept. In the present paper, both the potential and the limits of concrete foams were investigated, by voluntarily choosing concrete foams of high density which could not be crushed without increasing the solicitation. In fine, if its density is chosen accordingly to the potential threat, concrete foam is a potential energy-absorbing material for a cladding. However, its efficiency is low when compared to the more traditional honeycomb, and can only be counterbalance by its manufacturing process, which involves a foaming agent filling any given volume. Other limitations of the material are that its behavior is greatly dependent on numerous parameters: the specimen size and shape, the method of pore formation, direction of loading, age, water content, characteristics of ingredients used and the method of curing [29], cement–sand and water–cement ratios, curing regime, type and particle size distribution of sand and type of foaming agent used [30,31]. This may explain why such cladding is not often investigated and used.

On the other hand, the use of granular media involves several different mechanisms and parameters. Their strength depends first on the grain characteristic: the failure of these particles has been investigated for numerous granular material through the use of 1D compressive tests and Brazilian tests [31], while the particle geometry has been studied to understand the packing of several grains, known as the grain size distribution (GSD) [32,33,34], and the initial density of the granular media [35,36]. Both the porosity of the grain and the porosity of the medium are important. These parameters directly affect how the whole packing itself behaves under a given solicitation. If the stresses are high enough, grains can be crushed [37,38]. This is exactly what happens in this paper for the perlite which have been turned into dust, similarly to the concrete foam. In this case, dissipation through friction is assumed to be weak. On the contrary, if the stresses are insufficient, the overall response is dictated by inelastic rearrangement of the grains inside the volume [39]. If the displacement of the grain is possible, energy dissipation occurs through friction and deformation of the grains. In this paper, the grain splitting was mostly inexistent for the pumice, whose grains are not porous enough and more evenly distributed, increasing the density of the medium. It should be noted that the overall shape of the material still presents traces of damage, as if the external skin was torn off. This is a mark that the brittle collapse stress of the grains has not been reached, and most of the energy dissipation recorded is due to the friction between the grains. Contrary to the concrete foam where the crushing of the sample is necessary to dissipate energy, this is not the case for granular material. A comparison can be made with another granular media, sand. The porosity and grain size of sand is far lower than those of pumice, perlite and clay. For this reason, sand is the granular medium with the highest density and is often used as a confinement material to mitigate the detonation of an explosive. Pressure measurements made by Kirkpatrick [40] shows that despite having similar effects on the incident pressure, perlite was more efficient than sand to lower the reflected pressure. This indicates that despite having lower medium density, the higher grain porosity of the perlite leads to higher energy dissipation, even though the dissipation mechanisms in this study were not investigated. Bornstein also reached a similar observation with sands while using a panel [41]. The high grain density and low porosity of this granular medium lead to a low compressibility of the material, thus to a higher load transmission to the target: the behavior was closer to the one found in high density concrete.

In conclusion, energy dissipation is possible with granular media through grain deformation, grain splitting, and friction. The last point which differentiates the concrete foam and a granular media is momentum diffusivity. Like a fluid, granular media is able to spread momentum over a larger area, reducing the solicitation directly in front of the blast loading. In granular media, this happens because of the displacement of the grains. At the same time, a portion of the energy is also redirected and can even be extracted from the system. In the present experimental configuration, there is no boundary preventing the grains from sliding almost perpendicular to the solicitations, and the displacement of the grain are maximized, thus it must have an impact. A comparison can be made with the work of Langhorst [24] where the grains were either glued together using a spray, or placed in an epoxy resin. Langhorst stated that the best performances were achieved with the spray adhesive bound pumice, which presented an efficiency comparable to ours. There was no descriptions of the phenomenology at the time, but based on our recent observations, we can safely assume that this is a consequence of momentum diffusivity and energy extraction. The use of a plastic bag, or an adhesive glue, does not limit the displacement of the grains hence the spreading. Based on momentum conservation, it does not change the total momentum transmitted in the direction of the solicitation, and the load impulse reduction observed by Langhorst is most likely a consequence of his measurement technique. However, it does create radial momentum, expulsing grains and dust away, and thus redirecting energy which justifies most of Langhorst’s observation. This effect can be limited through at least three parameters: the granular media confinement, its grain porosity and its medium density. Regarding the confinement, Langhorst and the present study reported an important momentum spreading while Bornstein reported a total transmission of the momentum and energy with sand [41]. There was no momentum spreading for Bornstein, simply because the sand was confined inside a steel box. Regarding the porosity and the density, it is close to impossible to statute on its influence but it is safe to assume that it is related to friction of the grains. In this paper, it is possible to separate the source of energy dissipation between grain splitting and friction for the pumice and the perlite, both at the two extremes in terms of behavior. The perlite, where friction is neglected and the grains are pulverized, should present close to no radial spreading, contrary to pumice where important friction must also lead to spreading. For the clay, a granular material of intermediate density, there is a clear combination of both grains splitting and friction dissipations. The stress is high enough to break at least half of the particles (energy dissipation through grain splitting), while the debris and intact particles slides upon each other, (energy dissipation through friction and energy extraction through momentum spreading). When comparing these three brittle granular materials, clay seems to be the most efficient but it is also the sole media where the three phenomenon are present. Hydrogel would have been an interesting material to compare with the clay, as its elastic behavior tends to increase momentum spreading and energy redirection. Unfortunately, it is also the reason why the video recording is particularly inaccurate, as grains and dust are dispersed in the environment, preventing any analysis on the energy dissipation properties.

## 5. Conclusions

The explosive driven shock tube is an excellent tool to study the behavior of a sacrificial cladding. It has been used for many cellular materials, but the study of granular media highlights the limits of this approach. A macroscopic approach of the core behavior shows that granular media are also efficient energy dissipative materials, but it is close to impossible to quantitatively investigate how the energy is dissipated. The observations, coupled with the state of the art help understand the phenomenology but further investigations are necessary to confirm most of our assumptions. Especially, further studies are needed to ascertain the proportion of energy dissipation due to each phenomenon, or if it is even a consequence of energy transmission to the cladding as discussed in the methodology. Besides energy dissipation mechanisms, the displacement of the grain also allows a distribution of the momentum to the target over a larger area which could theoretically also reduce the deformation of the rear plate. Unfortunately, the free displacement of the grains, while being beneficial in practical application such as this one, is also not the main center of interest in blast protection studies.

## Figures and Tables

**Table 1:** Parameters computed for all tested crushable core.

Crushable Core		C4 (g)	Front Plate (g)	Densification	ε_d_ (-)	σ_0_ (kPa)	T (kJ·m^−3^)	E_abs_ (J)
Honeycomb 40 kg·m^−3^		50	652	Yes	0.71	661	564	282
		30	652	No	>0.60	626	>244	142
		15	652	No	>0.43	607	>133	67
Perlite 120 kg·m^−3^		50	652	Yes	0.47	631	294	147
		30	652	Yes	0.51	304	135	68
		15	652	Yes	0.35	222	79	39
Clay Ball 680 kg·m^−3^		50	652	No	>0.46	1881	>670	335
		30	652	No	>0.29	1404	>307	153
		15	652	No	>0.20	963	>170	85
Pumice 910 kg·m^−3^		50	652	No	>0.32	2262	>226	113
		30	652	No	>0.17	1880	>187	93
		15	652	No	>0.10	663	>49	25
Hydrogel 616 kg·m^−3^		50	652	Yes	n/a	734	n/a	n/a
		30	652	Yes	n/a	450	n/a	n/a
		15	652	No	n/a	159	n/a	n/a
Concrete foam	700 kg·m^−3^	50	652	No Crushing	Full transmission			
	400 kg·m^−3^	50	652	No Crushing	Full transmission			
	400 kg·m^−3^	50	224	No	n/a	2000	n/a	n/a
	150 kg·m^−3^	50	652	Yes	n/a	230	n/a	n/a

## Data Availability

Data is contained within the article.
